# Hepatopancreas-Specific Lectin Participates in the Antibacterial Immune Response by Regulating the Expression of Antibacterial Proteins

**DOI:** 10.3389/fimmu.2021.679767

**Published:** 2021-06-11

**Authors:** Xiao-Tong Cao, Xiao-Yi Pan, Meng Sun, Yan Liu, Jiang-Feng Lan

**Affiliations:** ^1^ Shandong Provincial Key Laboratory of Animal Biotechnology and Disease Control and Prevention, College of Animal Science and Veterinary Medicine, Shandong Agricultural University, Taian, China; ^2^ Key Laboratory of Healthy Freshwater Aquaculture, Ministry of Agriculture and Rural Affairs; Key Laboratory of Fish Health and Nutrition of Zhejiang Province; Zhejiang Institute of Freshwater Fisheries, Huzhou, China; ^3^ College of Fisheries, Huazhong Agricultural University, Wuhan, China

**Keywords:** C-type lectin, *Procambarus clarkii*, antimicrobial peptides, *Vibrio parahaemolyticus*, hepatopancreas

## Abstract

The hepatopancreas is an important digestive and immune organ in crustacean. There were low but stable numbers of microbes living in the hemolymph of crustacean, whereas the organs (including hepatopancreas) of crustacean were immersed in the hemolymph. It is very important to study the immune mechanism of the hepatopancreas against bacteria. In this study, a novel CTL (HepCL) with two CRDs, which was mainly expressed in the hepatopancreas, was identified in red swamp crayfish (*Procambarus clarkii*). HepCL binds to bacteria *in vitro* and could enhance bacterial clearance *in vivo*. Compared with the C-terminal CRD of HepCL (HepCL-C), the N-terminal CRD (HepCL-N) showed weaker bacterial binding ability *in vitro* and stronger bacterial clearance activity *in vivo*. The expression of some antimicrobial proteins, such as FLP, ALF1 and ALF5, was downregulated under knockdown of HepCL or blocked with Anti-HepCL after challenge with *Vibrio* in crayfish. These results demonstrated that HepCL might be involved in the antibacterial immune response by regulating the expression of antimicrobial proteins.

## Introduction

In the natural environment, vertebrates and invertebrates are exposed to various bacterial communities all the time, and their autoimmune systems play a key role in defending against pathogen infection (1–3). In vertebrates, innate immunity and adaptive immunity resist pathogen infection together. Invertebrates lack the typical antibody and T/B cell-based immune recognition system. The innate immune system of invertebrates plays an important role in defending against infectious agents (4–7). Pattern recognition receptors (PRRs) can trigger the innate immune response, such as Toll-like receptors and lectins (8–10). Among these PRRs, C-type lectins (CTLs) play an important role in the recognition of microbe-associated molecular patterns (MAMPs). In vertebrates, mannose-binding lectin (MBL), possesses a collagenous domain that can bind to the MBL-associated serine protease (MASP) to activate the complement system (11, 12).

CTLs have been characterized in many organisms, including vertebrates and invertebrates, and they primarily exert their function through the carbohydrate recognition domain (CRD) (13). Most CTLs contain a single CRD, and several of the CTLs have more CRDs. CTLs from invertebrates effectively participate not only in the recognition of pathogenic microbial glycans but also in antimicrobial functions, such as bacterial clearance, phagocytosis, cell adhesion, and prophenoloxidase activation (14–19). Fc-hsL with one CRD, a hepatopancreas-specific lectin, acts as a pattern recognition receptor in antibacterial defense (20). *Hc*Lec4 with four CRDs participate in antibacterial immune responses by regulating antimicrobial peptides (AMPs) expression in *Hyriopsis cumingii* (21). In *Anopheles gambiae*, two CTLs, CTL4 and CTLMA2 cooperate to exert anti-Gram-negative bacteria function (22). FcLec4 cooperates with *β*-integrin to promote hemocytic phagocytosis in *Fenneropenaeus chinensis* (19). It has been reported that CTLs could be involved in antifungal responses (23). Some CTLs have been reported to be directly or indirectly involved in the activation of the immune signaling pathways (24, 25). LvCTL1 possesses anti-white spot syndrome virus activity by binding to virus proteins in *Litopenaeus vannamei* (26). In contrast, some transmembrance C-type lectins promote *Mycobacterium tuberculosis* (27) and certain virus entry into host cells (28–31). CD45 phosphatase homolog recruits mosGCTL-1 to promote West Nile virus (WNV) infection in mosquitoes (32).

In crustaceans, especially shrimp, bacteria exist not only in the digestive tract but also in the hemolymph (33, 34). These bacteria possess a potential risk to shrimp farming. The hepatopancreas plays a key role in digestive and immune processes in shrimp. However, how shrimp restrain the proliferation of microbiota in the hepatopancreas needs to be further revealed. It has reported that CTL33 regulates intestinal homeostasis by mediating biofilm formation in *Marsupenaeus japonicas* (35). mosGCTLs binds gut microbiome and offset AMP activity to maintain gut microbiota homeostasis in *Aedes aegypti* (36). In this study, HepCL (GenBank No. MW727280), a novel CTL with two CRDs, mainly expressed in the hepatopancreas, was identified from red swamp crayfish (*Procambarus clarkii*). Bacterial clearance assays, bacterial binding assays and pathological sections were performed to analyze the role of HepCL in the antibacterial immune response in hepatopancreas. This study provides a new perspective for crustacean hepatopancreas resistance to bacterial infection.

## Materials and Methods

### *Vibrio* Challenge and Tissue Collection

Healthy red swamp crayfish (10-15 g) were obtained from a fish farm in Weishan, Shandong Province, China. These crayfish were acclimated in laboratory aquarium tanks with aerated freshwater at 22°C for one week before being involved in this study. Organs (hemocytes, hepatopancreas, gills, stomach and intestine) were collected from at least three crayfish for further analyses, and total RNA was extracted with RNAiso Plus (Takara, China). For hemocyte collection, hemolymph was extracted with a syringe containing 1 ml cold anticoagulant buffer [0.14 M NaCl, 0.1 M glucose, 30 mM trisodium citrate, 26 mM citric acid, and 10 mM ethylene diamine tetra acetic acid (EDTA), pH 4.6] at 4°C (37) and immediately centrifuged at 800 g for 5 min (4°C). For bacterial challenge assays, each crayfish was injected in the abdomen with 25 μl of *Vibrio parahaemolyticus* (1 × 10^7^ CFU in PBS). The total RNA and protein of the hepatopancreas were separately extracted from 10 healthy crayfish and collected at 12 h post injection (hpi). cDNA was synthesized by using the PrimeScript RT-PCR Kit (Vazyme, China) for quantitative real-time PCR (qRT-PCR) analysis. The assay was performed in triplicate.

### Expression and Purification of Recombinant HepCL

In the experiment on prokaryotic recombinant expression, primers (HepCL-EX-F/R, HepCL-N-EX-F/R, HepCL-C-EX-F/R, **Table 1**) were used to amplify fragments of HepCL (957 bp), HepCL-N (345 bp), and HepCL-C (519 bp). PCR was programmed at 95°C for 5 min, 35 cycles at 95°C for 30 s, 58°C for 30 s, 72°C for 50 s, and one cycle at 72°C for 10 min. The DNA fragments were linked to the vector pGEX-4T-1. Recombinant HepCL, HepCL-N, and HepCL-C were expressed in *Escherichia coli* (*E. coli*) BL21 cells (TransGen, China). The recombinant proteins were purified by affinity chromatography using GST resin (Sangon, China) following the method described in previous papers (38). HepCL polyclonal antibodies were prepared by the company using recombinant proteins (Frdbio, China).

**Table 1:** Primers used in this study.

Primers	Sequences (5’-3’)
HepCL-EX-F	TACTCAGGATCCATGGACTGTCCCTAC
HepCL-EX-R	TACTCACTCGAGCTA GGT AGC CATGCA
HepCL-N-EX-F	TACTCAGGATCCATGGACTGTCCCTAC
HepCL-N-EX-R	TACTCACTCGAGTTA AAATTCGCATAA
HepCL-C-EX-F	TACTCAGGATCCATGGCGTGTGAGGCA
HepCL-C-EX-R	TACTCACTCGAGCTAGGTAGCCATGCA
HepCL-RT-F	GGTTTAGTGCTGGACGGAGTT
HepCL-RT-R	CTGGGCGACATCATAGGTG
Lys-i1-RT-F	GTCAACCCACCCTCAATAAC
Lys-i1-RT-R	CTTGTGAATCAGGGCGTA
ALF1-RT-F	GAAGCGATGACGAGGAGCAAT
ALF1-RT-R	GACGGGTTGGCACAAGAGC
ALF2-RT-F	CAAACTGGGCGGGTTATGG
ALF2-RT-R	TGACGAAGTCCCTGGTGGC
ALF5-RT-F	GGGGAGGTGAGGCTACT
ALF5-RT-R	TTCCTGCTCGGTGATG
FLP-RT-F	CGACAAGACCGTCAAGGC
FLP-RT-R	ATCTGTGTTCGTTTTTTATTTT
18S-RT-F	TCTTCTTAGAGGGATTAGCGG
18S-RT-R	AAGGGGATTGAACGGGTTA
HepCL-RNAi-F	GCGTAATACGACTCACTATAGG ACGACCAAGCAATAGAAA
HepCL-RNAi-R	GCGTAATACGACTCACTATAGG CACAGGGAATTAAATCAAAC
GFP-RNAi-F	GCGTAATACGACTCACTATAGGTGGTCCCAATTCTCGTGGAAC
GFP-RNAi-R	GCGTAATACGACTCACTATAGGCTTGAAGTTGACTTGATGCC

### Tissue Distribution and Expression Profiles

The tissue distribution of HepCL in normal crayfish was analyzed by qRT-PCR and western blot. For qRT-PCR, a pair of primers (HepCL-RT-F/R, **Table 1**) was used to detect the transcriptional levels of HepCL with 18S rRNA (with primers 18S-RT-F/R, **Table 1**) as an internal control. The qRT-PCR procedure was as follows: 94°C for 2 min, 40 cycles at 94°C for 15 s and 60°C for 30 s. The results were analyzed by using the 2^-ΔΔCt^ method (39). Sodium dodecyl sulfate polyacrylamide gel electrophoresis (SDS-PAGE) was used to analyze protein samples, and then the proteins were transferred onto nitrocellulose (NC) membranes followed by blocking in nonfat milk (4% in TBS: 150 mM NaCl, 10 mM Tris-HCl, pH 7.4) for 2 h. The NC membranes were incubated overnight with primary antibody (rabbit anti-HepCL or *β*-actin antiserum), washed three times with TBST (0.02% Tween 20 in TBS) and then with TBS. The specific polyclonal antiserum of HepCL and *β*-actin were prepared in our lab by immunizing rabbit with the purified GST-HepCL and His-actin. The NC membrane was incubated with HRP goat anti-rabbit IgG (1/10,000 diluted in TBS) (Proteintech, China) for 1 h. After washing with TBST and TBS as above, the protein bands were detected using ChemiLuminescence (CWbio, China). qRT-PCR and western blot were also used to detect HepCL expression patterns after *Vibrio* infection following the methods described above.

### RNA Interference Assay

The specific primers HepCL-RNAi-F/R and GFP-RNAi-F/R (**Table 1**) were used in this assay. A commercial transcription T7 kit (Thermo, USA) was used to synthesize dsRNA following a previously reported method (40). Crayfish were divided into three groups (3 crayfish/group) and injected with dsHepCL (20 μg) or dsGFP. The normal group was the group of unchallenged crayfish. Total RNA from the hepatopancreas was extracted to evaluate the RNAi efficacy at 48 h after the injection of dsRNA.

### Bacterial Clearance Assay

Crayfish were divided into two groups (3 crayfish/group) and injected with 50 μg of (1 μg/μl) HepCL. GST-Tag was used as a control. One hour after injection, the crayfish were challenged with 25 μl *Vibrio* (1 × 10^9^ CFU/ml). Thirty minutes after bacterial injection, the hemolymph of each crayfish was collected, and 50 μl of the hemolymph was cultured on solid Luria-Bertani (LB) plates at 37°C overnight. The numbers of bacteria on each plate were counted. HepCL was knockeddown *in vivo*, and then the bacterial clearance assay was performed as above. The experiment was repeated three times.

To study the role of the different domains of HepCL in antibacterial immune responses, crayfish were divided into four groups (3 crayfish/group) and injected with 50 μg of (1 μg/μl) HepCL, HepCL-N, or HepCL-C protein. GST-Tag was used as control. The experiment was carried out as above.

### Survival Rate Assay

To analyze the function of HepCL, crayfish (60 crayfish per group, 10-15 g) were divided into two groups to evaluate the crayfish survival rate. The experimental group was injected with recombinant HepCL (50 μl, 1 μg/μl) and then challenged with *Vibrio* (25 μl 1 × 10^7^ CFU/ml in PBS) within 1 h after the first injection. GST-Tag was used as a control. The number of dead crayfish was monitored every day, and the cumulative survival rates of the two groups of crayfish were calculated.

### Pathological Analysis of the Hepatopancreas After Challenge with *Vibrio*

Crayfish were divided into six groups (6 crayfish per group), one group was not treated as control, and 50 μg of HepCL, HepCL-N, HepCL-C or GST-Tag protein were injected into other five group crayfish. Overnight-cultured *Vibrio* or heat-inactivated *Vibrio* were washed three times with PBS and diluted to 10^7^ CFU/ml, and then, 50 μl *Vibrio* or heat-inactivated *Vibrio* was injected into each crayfish 1 h after protein injection. Hepatopancreases were collected after 24 hpi and fixed with 4% paraformaldehyde solution. Then, all samples were sent to the company (Google, China) for pathological sections, then pathological sections of hepatopancreas were observed and analyzed under microscope in our lab.

### Bacterial Binding Assay

Six kinds of bacteria were involved in the bacterial binding assay (gram-positive bacteria: Staphylococcus aureus, Bacillus subtilis and Streptococcus agalactiae; and gram-negative bacteria: Edwardsiella piscicida, V. parahaemolyticus and Aeromonas hydrophila). All bacteria were cultivated overnight, harvested at 3000 rpm for 10 min by centrifugation, and then washed three times with PBS. The HepCL, HepCL-N, HepCL-C or GST-Tag proteins were mixed and incubated with different bacteria (10^7^ CFU/ml) respectively at 37°C for 30 min at a final concentration of 50 μg/ml. The samples were centrifuged at 3000 rpm for 10 min and washed three times with PBS. Finally, bacterial pellets in each tube were resuspended in 20 μl of PBS and boiled with 2 × loading buffer for 5 min. Western blot was used to determine the binding activity of HepCL, HepCL-N, and HepCL-C to the bacteria with anti-GST-Tag (Cwbio, China).

### Expression Analysis of Antibacterial Proteins After Blocked HepCL or HepCL Was Knockeddown

The expression levels of antibacterial proteins were detected at 6 h after *Vibrio* or *S. aureus* infection in the hepatopancreas of crayfish. The expression levels of FLP, ALF1, ALF2, ALF5 and Lys-i1 were detected by qRT-PCR with specific primers (**Table 1**). After blocked HepCL with Anti-HepCL, the expression levels of antibacterial proteins were detected at 6 h after challenge with *Vibrio* in the hepatopancreas of crayfish. Anti-actin was used as control. The assay was repeated three times.

To further verify the role of HepCL in regulation the expression of AMPs, it was knockeddown. And then the expression levels of antibacterial proteins were detected in hepatopancreas of HepCL-knockdown crayfish at 6 h post *Vibrio* challenge. dsGFP was used as control. The assay was repeated three times.

## Results

### Tissue Distribution and Expression Profiles of HepCL

**Figure 1:**
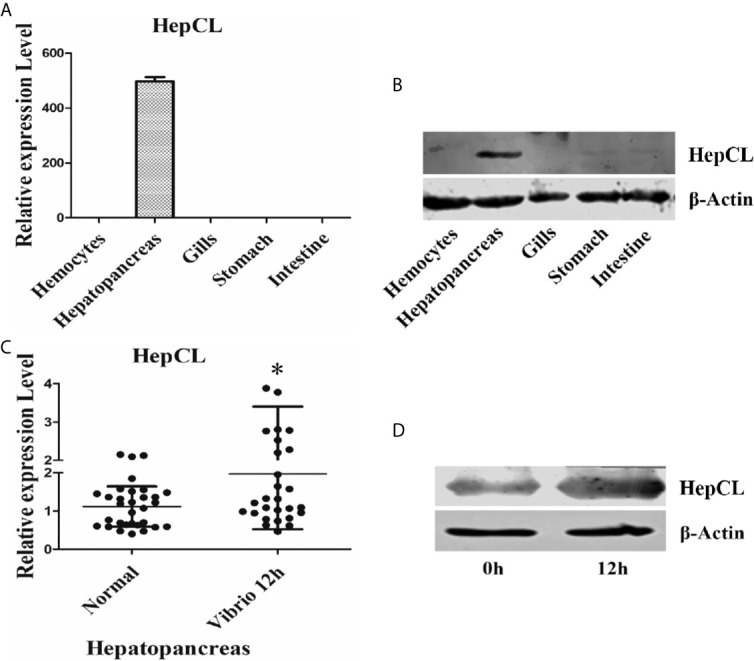
Tissue distribution and expression profile of HepCL. **(A, B)** The tissues distribution of HepCL was detected by qRT-PCR and western blot. **(C, D)** Expression profile of HepCL were analyzed after challenged with *Vibrio* at 12 h also detected by qRT-PCR and western blot in hepatopancreas. 18S rRNA (mRNA level) and β-Actin (protein level) were used as the control. The asterisk represented the significant difference, p < 0.05.

The tissue distribution of HepCL was analyzed in hemocytes, hepatopancreas, gills, stomach and intestine by qRT-PCR and western blot. HepCL was observed mainly in the hepatopancreas at the mRNA level (**Figure 1A**). HepCL was mainly expressed in the hepatopancreas at the protein level, and it was also detected in the stomach and intestine (**Figure 1B**).

Expression patterns of HepCL after challenge with *Vibrio* were analyzed with qRT-PCR and western blot. The results showed that the expression level of HepCL was upregulated at 12 h after *Vibrio* challenge in the hepatopancreas (**Figures 1C, D**).

### HepCL Influence Survival Rate and Bacterial Clearance

**Figure 2:**
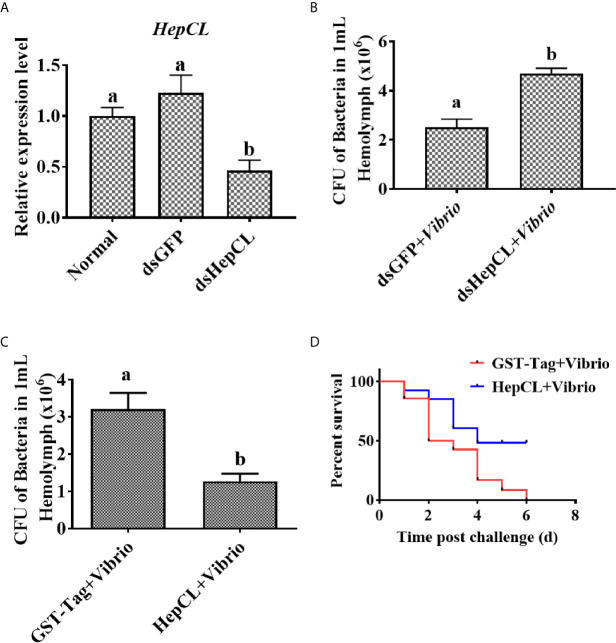
HepCL influence survival rate and bacterial clearance capacity of crayfish. **(A)** After HepCL was knocked down, bacterial clearance experiments were performed. qPCR was used to analyze the interference effect of HepCL. **(B)** After hep was knocked down, the residual bacteria in crayfish were counted, dsGFP was used as control. **(C)** The bacterial clearance capacity of the crayfish was analyzed after HepCL was injected into Crayfish, GST- Tag protein was used as control. **(D)** The survival rate was counted after HepCL and *Vibrio* injected, GST-Tag was used as the control. Differences among the groups were analyzed using a t-test, different letters indicate significant differences p < 0.05.

To further study the role of HepCL in the anti*Vibrio* immune response *in vivo*, survival assay and bacterial clearance assay were performed. The results showed that after knockdown of HepCL (**Figure 2A**), the number of bacteria in dsHepCL injection crayfish increased significantly compared with that in the control crayfish (**Figure 2B**). As shown in **Figure 2C**, the number of bacteria in HepCL injection crayfish decreased significantly compared with that in the control crayfish. The survival assay showed that the crayfish began to die on the first day after injection. All of the crayfish in the GST-Tag injection group died within six days, whereas half of the crayfish survived in the HepCL injection group at six day after injection (**Figure 2D**). These results suggest that HepCL improved the survival rate of crayfish and enhanced bacterial clearance in crayfish.

### Histological Section Analysis

**Figure 3:**
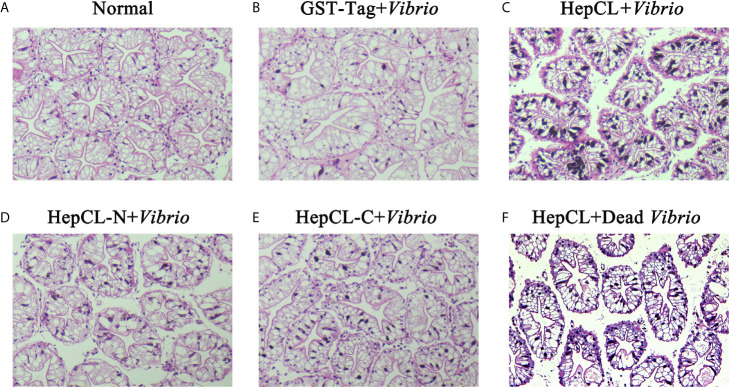
Histological section analysis of hepatopancreas. H&E stain was used in this assay. Proteins (GST-Tag **(B)**, HepCL **(C)**, HepCL-N **(D)**, HepCL-C **(E)** were injected into crayfish, then crayfish was challenged with 10^6^ *Vibrio.* **(F)** After protein HepCL injection, the crayfish was injected with heat-inactivated *Vibrio* (Dead *Vibrio*). Normal crayfish was used as control **(A)**.

To further analyze the role of HepCL in the hepatopancreas, GST-Tag+ *Vibrio*, HepCL+*Vibrio*, HepCL-N+*Vibrio*, HepCL-C+*Vibrio* and HepCL+Dead *Vibrio* were injected into crayfish. The hepatopancreas tissue of each crayfish was fixed for histological sectioning at 24 hpi. As shown in **Figure 3B**, vacuolization in the hepatopancreas was found, and the connection between the lobules of the hepatopancreas was broken after *Vibrio* infection. The pathological tissue morphology of each crayfish injected with HepCL+*Vibrio* (**Figure 3C**), HepCL-N+*Vibrio* (**Figure 3D**) or HepCL+Dead *Vibrio* (**Figure 3F**), revealed shrinkage and a cell infiltration inflammatory response in the hepatopancreas compared with that of control and injected HepCL-C+ *Vibrio* (**Figure 3E**) crayfish. Obvious tissue damage was present in the hepatopancreas of *Vibrio*-infected crayfish. The hepatopancreas of crayfish injected with HepCL showed a stronger inflammatory response than those injected with other proteins.

### Influence of the Different Domains of HepCL on Bacterial Clearance

**Figure 4:**
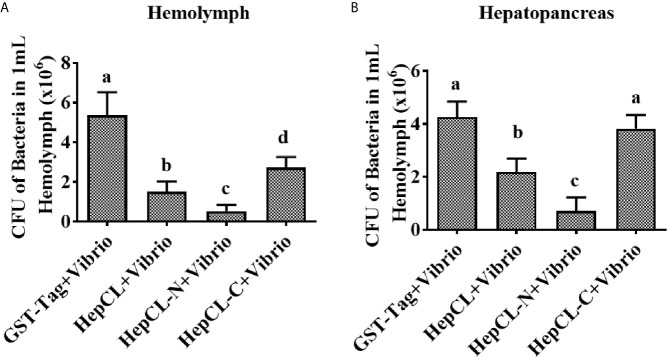
Bacterial clearance assay. Bacterial clearance assay was involved to study the function of different domain of HepCL in antibacterial immunity. Crayfish were injected with HepCL, HepCL-N, HepCL-C 1 h later challenged with *Vibrio*, after 30 mpi, hemocytes **(A)** and hepatopancreas **(B)** were collected and the number of bacteria was counted. The group of GST-tag was used as control. Differences between groups were analyzed using one-way analysis of variance (ANOVA). Different letters indicate significant differences (p < 0.05).

To identify the role of the two domains of HepCL in the antibacterial immune response. Bacterial clearance was observed in the hemolymph and hepatopancreas after HepCL, HepCL-N or HepCL-C protein injection. Crayfish were injected with the corresponding proteins and then infected with *Vibrio* 1 h after the first injection. Thirty minutes after infection, hemolymph and hepatopancreas homogenates were collected. The bacterial clearance activity of crayfish injected with HepCL or HepCL-N was more effective than those crayfish injected with HepCL-C in both the hemolymph (**Figure 4A**) and hepatopancreas (**Figure 4B**). These results suggest that N-terminal CRD (HepCL-N) plays a key role in maintaining bacterial homeostasis.

### Bacterial Binding Analysis

**Figure 5:**
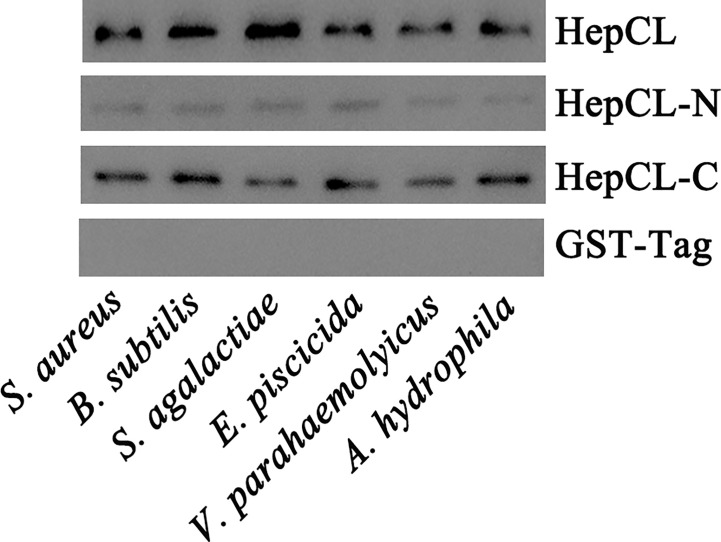
Bacterial binding assay. Six kinds of bacteria *S. aureus, B subtilis and S. agalactiae, E piscicida, V. parahaemolyticus and A hydrophila*, were used to analyze the binding activity of recombinant HepCL, HepCL-N, HepCL-C by western blot, and detected with anti-GST-Tag antibody. GST-Tag was involved as control.

It has reported that CTLs could bind the bacteria, then promote phagocytosis or inhibit bacteria proliferation. To gain further insight into the role of HepCL in antibacterial immunity, the bacterial binding activities of HepCL, HepCL-N and HepCL-C were analyzed by western blot using the six kinds of bacteria. As shown in **Figure 5**, HepCL, HepCL-N and HepCL-C could bind to all the tested bacteria, while the binding activity of HepCL-N was weaker than that of HepCL and HepCL-C. These results suggest that HepCL-C contributed more bacterial binding activity.

### HepCL Regulates the Expression of Antimicrobial Proteins

**Figure 6:**
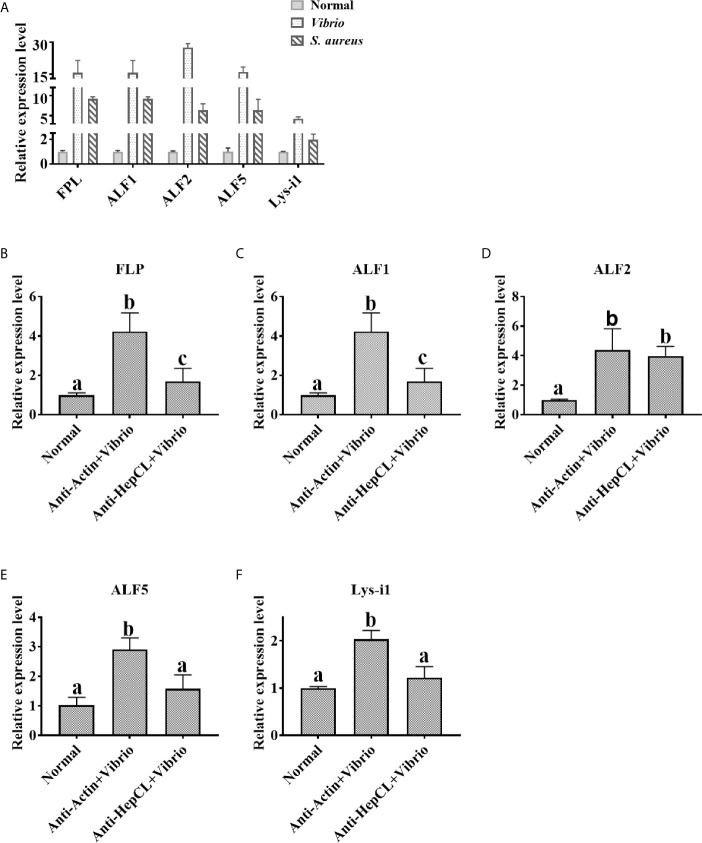
The expression levels of antimicrobial proteins after Anti-HepCL-*Vibrio* injection. **(A)** The expression levels of FLP, ALF1, ALF2, ALF5 and Lys-i1 were analyzed by qRT-PCR after *Vibrio* and *S. aureus* infection. **(B–F)** The expression levels of FLP, ALF1, ALF2, ALF5 and Lys-i1 was analyzed when blocked HepCL with Anti-HepCL after *Vibrio* infection, Anti-actin was used as control. Differences between groups were analyzed using one-way analysis of variance (ANOVA). Different letters indicate significant differences p < 0.05.

**Figure 7:**
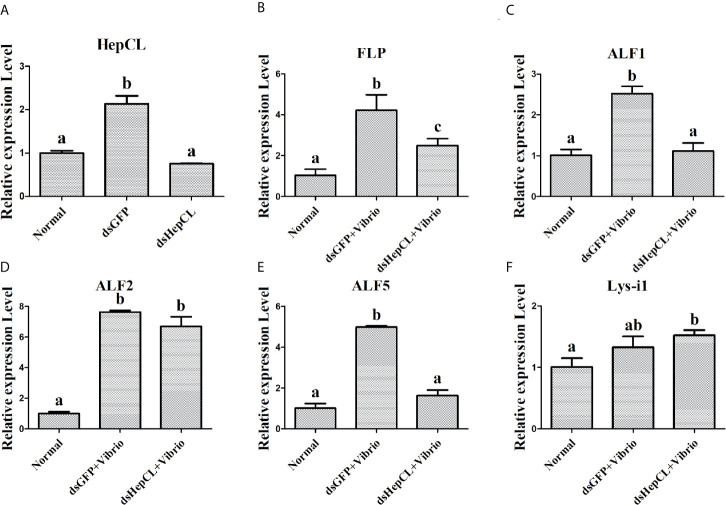
The expression levels of antimicrobial proteins after knockdown of HepCL. **(A)** qPCR was used to analyzes the effect of HepCL was knockeddown. **(B–F)** The expression levels of FLP, ALF1, ALF2, ALF5 and Lys-i1 was analyzed under knockdown of HepCL after *Vibrio* infection by qPCR, dsGFP was used as control. Differences between groups were analyzed using one-way analysis of variance (ANOVA). Different letters indicate significant differences p < 0.05.

HepCL did not show any activity in inhibiting bacterial proliferation (data not shown). We wanted to know whether HepCL is involved in anti-bacterial immunity as a regulatory factor. We analyzed whether HepCL regulates the expression of antimicrobial proteins. The antimicrobial proteins expression levels were detected after *Vibrio* and *S. aureus* infection. The result showed that the expression levels of all antimicrobial proteins which were tested, were significantly upregulated (**Figure 6A**). To analyze the possible mechanism of HepCL in antibacterial immunity, the expression levels of antimicrobial proteins were analyzed, in Anti-HepCL injection- or HepCL knockeddown-crayfish at 6 h after *Vibrio* challenge (**Figure 7A**). The expression levels of five antimicrobial proteins were evaluated, including four antimicrobial peptides (ALF1, ALF2, ALF5 and Lys-i1) and a ficolin-like protein (FLP), after *Vibrio* challenge in crayfish. The results showed that in Anti-HepCL injection crayfish the expression levels of FLP, ALF1, ALF5 and Lys-i1 were downregulated (**Figures 6B, C, E, F**), while the expression levels of ALF2 was not (**Figure 6D**) compared with those in the Anti-actin injection crayfish. In dsHepCL injection crayfish, the expression levels of FLP, ALF1 and ALF5 were downregulated (**Figures 7B, C, E**), while the expression levels of ALF2 and Lys-i1 were not (**Figures 7D, F**) compared with those in the dsGFP injection group. All the above results indicated that block and knockdown of HepCL specifically downregulated the expression of some antimicrobial proteins.

## Discussion

Invertebrates possess an innate immune system, lack acquired immune system that is dependent on immunoglobulin (41). Pathogen-associated molecular patterns (PAMPs) are detected by host pattern recognition receptors (PRRs) in innate immunity. C-type lectins are important pattern recognition receptors in the innate immune system of invertebrates. Lectins are generally expressed in lymphocytes and act as immunoglobulins (42, 43). Some insect lectins can bind to bacterial lipopolysaccharides by O-specific chains (44). A hepatopancreas specific C-type lectin (*Pm*LT) binds to bacteria and activates the innate immunity of *Penaeus monodon* (45). CTL33, mainly in stomach and intestine, mediated biofilm formation by intestinal bacteria was reported, providing a new view into the homeostasis of intestinal regulation in invertebrates (35). MjHeCL inhibits the proliferation of the hemolymph microbiota in maintaining a healthy status (18). The hepatopancreas is one of the major digestive and immune tissues of shrimp. In this study, we identified a C-type lectin from crayfish, HepCL, with two different CRDs that was primarily expressed in the hepatopancreas and plays a very important role in the crayfish antibacterial immune response. HepCL was primarily expressed in the hepatopancreas at mRNA and protein level, and it was detected low expression at protein level in stomach and intestine (**Figures 1A, B**). There is a signal peptide at the N-terminus of HepCL. These results suggest that HepCL may be a secretory protein that is synthesized from the hepatopancreas and secreted into circulatory hemolymph.

Previous studies have shown that most CTLs play a key role in immune responses, and their expression levels can be upregulated after challenge by pathogenic microorganisms. *Vibrio* infection could cause pathological changes in the hepatopancreas of shrimp. Ficolin-like protein (FLP1), a type of lectin, could protect the connection between the lobule from *Vibrio* infection in crayfish (46). As shown in **Figure 3B**, *Vibrio* infection induced an obvious inflammatory response in the hepatopancreas. There were obvious hepatopancreatic tube shrinkage and cell infiltration when injected with HepCL and HepCL-N, compared with that which injected with GST or HepCL-C (**Figure 3**). STAT3 signaling hyperactivation occurs in most human cancers and is connected with a poor prognosis (47). These results suggest that HepCL may be closely related to the activation of inflammatory response.

The histopathological analysis prompted us to consider the possibility that HepCL may be connected with other immune responses. CRDs usually contain key motifs, such as EPN (Glu-Pro-Asn), EPS (Glu-Pro-Ser), LND (Leu-Asn-Asp), and QAP (Glu-Ala-Pro), which bind to polysaccharides (9, 48). HepCL contains two dissimilar CRDs. HepCL and the C-terminal CRD (HepCL-C) showed strong binding activity, but the N-terminal CRD (HepCL-N) with “EPS and LND” motifs was weak (**Figure 5**). The bacterial clearance efficiency of the HepCL-N injection crayfish was higher than that of the HepCL-C injection crayfish (**Figure 4**). CfLec-3 from scallop with three CRDs that occurred obvious different functions, as CRD1 showed strong binding activity, while CRD2/3 showed more function in facilitating hemocyte mediated opsonisation (49). Our results suggest that other molecules which may be regulated by HepCL, rather than CRDs itself, play a direct antimicrobial role. Thus, we speculate that the N-terminal CRD (HepCL-N) may trigger the innate immune response, while the C-terminal CRD (HepCL-C) plays a role in the recognition of MAMPs.

Previous studies showed that Ab and MBL are classical opsonins in mammals, and in invertebrates, some CTLs were also reported as opsonins, pattern recognition or effector molecules to exert immune functions (50–52). In the current study, we found that HepCL could bind to bacteria and enhance bacterial clearance activity in crayfish, but it did not inhibit bacterial proliferation *in vitro* (data not shown). Further study showed that HepCL could regulate the expression of antimicrobial proteins (**Figures 6**, **7**). Invertebrates lack an acquired immune system, and effector molecules such as antimicrobial peptides (AMPs) play important roles in innate immunity (53). The rapid synthesis and release of active AMPs is a significant strategy in invertebrate host defenses. In *M. japonicas*, *Mj*CC-CL upregulates the expression of AMPs *via* the JAK/STAT signaling pathway in the antibacterial response (24). HcLec4 is involved in the regulation of AMP expression (21). MjHeCL binds to hemocytes to modulate the expression of AMPs (18). A new AMP inhibits *A. hydrophila* infection in crayfish, and new insights into the maturation of AMPs were revealed (54). We speculated that HepCL may modulate the expression of antimicrobial proteins to exert an antibacterial immune response.

Previous studies showed that Fibrinogen-related proteins are mainly function as PRRs in invertebrates and vertebrates (55, 56). Two ficolin-like proteins function as pattern recognition receptors in the innate immunity of crayfish (57). A Fibrinogen-related protein (FREP) in *M. japonicus* was reported that plays an important role in the antibacterial immunity by binding bacteria and enhancing bacterial clearance (58). Our previous results showed that ficolin (FLP) could bind to bacteria and inhibit the replication of bacteria in *P. clarkii* (46). Some shrimp ALFs, which is a type of AMPs, have important motifs interact with LPS in antibacterial immunity (mainly Gram-negative bacteria) (59). ALF5 inhibits proliferation of microbiota by binding to RPS4 and MscL of *E. coli* in Crayfish (60). In this project, the expression levels of two AMPs (ALF1 and ALF5) and FLP were upregulated in *Vibrio* and *S. aureus* infection crayfish (**Figure 6A**), while the expression levels of them were downregulated after challenge with *Vibrio* in Anti-HepCL injection crayfish (**Figure 6**) and HepCL knockeddown crayfish (**Figure 7**). When the crayfish was injected with HepCL protein, the number of bacteria in the crayfish decreased than those in control crayfish (**Figure 2C**), while in the crayfish of HepCL was knockeddown, the number of bacteria in the crayfish increased than those in control crayfish (**Figure 2B**). The present study revealed that HepCL might be involved in the antibacterial immune response by regulating the expression of antimicrobial proteins rather than by the classical inhibition of bacterial replication and decreasing pathological changes. Hyperactivation of the immune system (overly strong or long-lasting activation) can subsequently lead to organ dysfunction and increased susceptibility to secondary infections (61). An inflammatory storm caused by the high expression of antimicrobial molecules in a short period of time leads to cell infiltration. The underlying mechanism by which HepCL regulates the expression of antibacterial proteins needs further study.

## Data Availability Statement

The datasets presented in this study can be found in online repositories. The names of the repository/repositories and accession number(s) can be found below: https://www.ncbi.nlm.nih.gov/genbank/, 2437240.

## Author Contributions

All authors listed have made a substantial, direct, and intellectual contribution to the work, and approved it for publication.

## Funding

This work was supported by grants from the open project of Agriculture Ministry Key Laboratory of Healthy Freshwater Aquaculture (No. ZJK201804), the National Key Research and Development Program of China (No. 2020YFD0900303), Technical Innovation Project of Hubei province (No. 2018ABA103), Research on Public Welfare Technology Application Projects of Zhejiang Province (No. 2017C32012).

## Conflict of Interest

The authors declare that the research was conducted in the absence of any commercial or financial relationships that could be construed as a potential conflict of interest.
